# A forgotten complication of diaper dermatitis: Granuloma gluteale infantum

**DOI:** 10.4103/1319-1683.71994

**Published:** 2010

**Authors:** Nadya A. Al-Faraidy, Sahar H. Al-Natour

**Affiliations:** *Department of Dermatology, College of Medicine, University of Dammam, Dammam, Kingdom of Saudi Arabia*

**Keywords:** Granuloma gluteale infantum, fluorinated steroids, diaper dermatitis

## Abstract

Granuloma Gluteale Infantum (GGI) is a rare condition of unclear etiology,[1] presenting as asymptomatic cherry red nodules in the diaper area appearing in the setting of primary irritant contact dermatitis.[2] A 50 day old infant with GGI is presented to emphasize that the condition may be easily missed, and that it may result from the misuse of fluorinated topical steroids used to treat a rash in the diaper area. This is the first case reported from Saudi Arabia.

## INTRODUCTION

Granuloma gluteale infantum (GGI) was described as early as 1891 and was later named “vegetating bromidism” due to its occurrence on application of bromide containing ointments.[3] Since Tappeiner and Pfleger first reported 6 cases,[4] similar cases have been reported in the USA, Europe and Japan.[2]

## CASE REPORT

**Figure 1:**
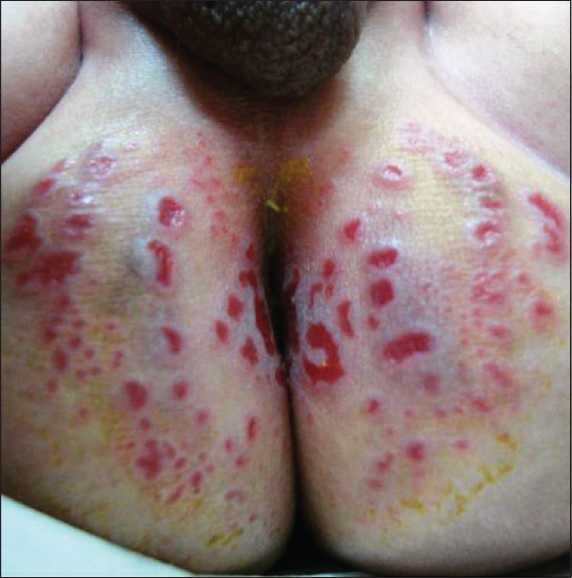
The patient initially presented with multiple purple-red nonoozing indurated nodules on the gluteal area.

A 50- day old Saudi male presented to our dermatology clinic with a worsening rash in the diaper area of 5 weeks duration. At the onset of the rash, he was treated with a topical nystatin and triamcinolone acetonide cream for 2 weeks with no improvement. He then, was given a cream containing zinc oxide and hydrocortisone butyrate 0.1% with minimal improvement. At this time, he presented to our clinic where a diagnosis of “eroded diaper dermatitis” was made. He was treated with topical fusidic acid and betamethasone valerate 0.1% creams. In one week, the rash had resolved but multiple deep purple-red nodules, 0.5-1 cm in size were noted on the convexities of the gluteal region with the long axis of the nodules parallel to the skin folds. The nodules were indurated, non-oozing and not tender [Figure 1]. A clinical diagnosis of GGI was made and a biopsy was performed to rule out other conditions. Histological examination revealed prominent parakeratosis overlying regular acanthosis. There was mild to moderate spongiosis and exocytosis and a moderate mononuclear perivascular infiltrate. PAS and Grocott stains did not show any fungal elements [Figures 2a,b]. The nodules resolved completely with slight hyperpigmentation and residual atrophic scarring within one month after discontinuation of the topical fluorinated steroids [Figure 3].

**Figure 2a:**
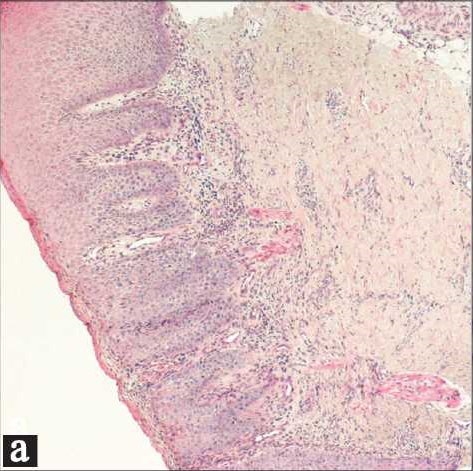
Histology showed parakeratosis, moderate regular acanthosis, moderate spongiosis and mild exocytosis. (H and E stain ×50)

**Figure 2b:**
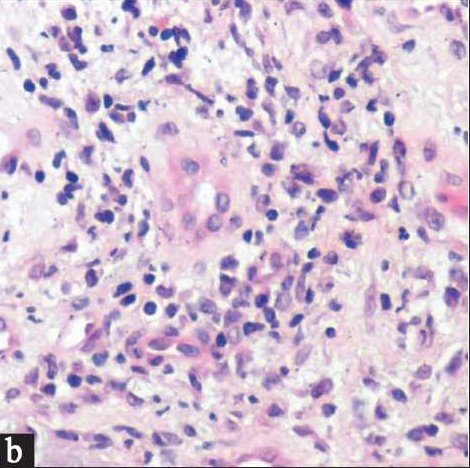
Moderate predominantly mononuclear perivascular mixed inflammatory dermal cell infiltrate. (H and E stain ×400)

**Figure 3:**
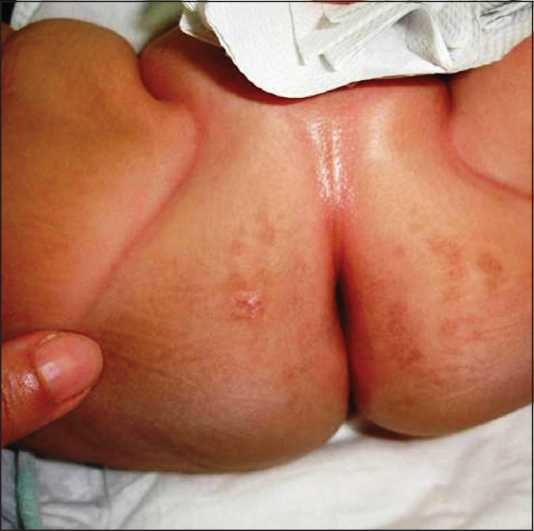
Resolution of the nodules with slightly atrophic hyperpigmented scarring 1 month after stopping the fluorinated steroids.

## DISCUSSION

Diaper dermatitis is the most common skin condition in infants, and accounts for 20% of all dermatitides in that age group with no predilection to a certain age or gender. The average onset is at 9-12 months of age, but it may occur as early as the first month. Diaper dermatitis resolves spontaneously with empirical treatment in most patients. Few patients present with complications which include punched out ulcers or erosions with elevated borders typical of Jacquet’s erosive diaper dermatitis; pseudoverroucus papules and nodules, and 0.5-4.0 cm asymptomatic cherry red plaques and nodules of granuloma gluteale infantum.

Granuloma gluteale infantum is a rare though a distinctive dermatosis of infancy of unknown etiology.[1] Irritant contact dermatitis,[5] candidiasis,[2] occlusion from diapers in incontinent infants and adults[6,7] and the prolonged use of benzocaine[8] and fluorinated steroids[2,9] have been considered as contributing factors for the development of GGI.

The histological picture is that of a non-specific perivascular dermal infiltrate composed of neutrophils, lymphocytes, histiocytes, plasma cells and eosinophils; with an overlying hyperplastic epidermis and parakeratosis.

Our case fits the classic description of GGI as far as the clinical presentation and histologic features and occurrence after prolonged use of fluorinated steroids. Attention is drawn to this condition as the appearance of the dermatosis can be suggestive of granulomatous or neoplastic diseases.[5] When recognized, GGI responds rapidly to removal of possible aggravating factors such as topical fluorinated steroids as the case in our patient leaving hyperpigmentation and slight atrophic scarring.[9–11]

The diagnosis of GGI should be considered in longstanding and unresponsive cases of diaper dermatitis in infants as the inflammation can easily mask the underlying characteristic nodules suggestive of the diagnosis. The main conditions that can be mistaken for GGI with the differentiating features are summarized in Table 1.

**Table 1:** Conditions that can be mistaken for GGI with differentiating features

Differential diagnosis of GGI	Differentiating features
Jacquet’s erosive dermatitis	Tender punched out painful ulcers in ages above 6 months, resulting from lack of hygiene and infrequent diaper changing, no use of medications
Allergic contact dermatitis	Erythematous papules and patches resolving usually with empirical treatment of steroids rather than persisting
Candidiasis	Erythematous patches favoring the folds, with scattered satellite lesions, positive culture, responding to antifungal
Tinea corporis	Usually single or few annular lesions(but maybe plaques) with scales. KOH shows fungal elements, cultures may be positive, responds well to antifungals
Psoriasis	Erythematous plaques in the diaper area. May have positive family history of psoriasis, responds partially to empirical steroids
Seborrheic dermatitis	Other common sites involved would be the scalp(cradle cap) and eyes brows.
Langerhans histiocytosis	Unwell infant, organomegaly, seborrheic dermatitis like lesions on the scalp, persistent diaper rash, petechia, pancytopenia, diagnostic biopsy(histiocytic infiltrate)
Secondary syphilis	May present as puched out ulcers, papules or plaques, as well as condyloma accuminata. FTA Abs positive
